# Supplementary figures and images for: Symptom burden, viral load, and antibody response to ancestral SARS-CoV-2 strain [D614G] in an outpatient household cohort
Source: PLoS One. 2026 Feb 5;21(2):e0313467. doi: 10.1371/journal.pone.0313467 (PMC12875513; doi:10.1371/journal.pone.0313467)

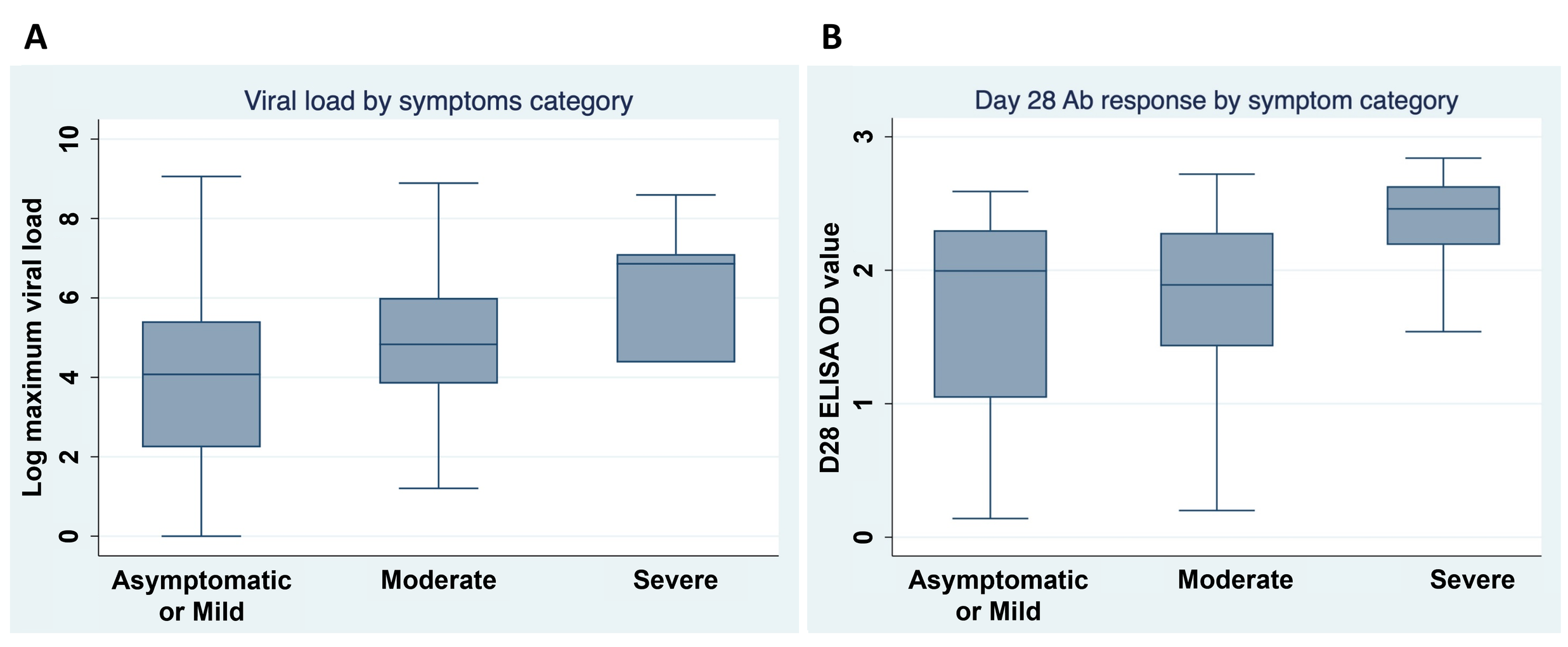

Supplement: S1 Fig — (TIF) [file pone.0313467.s001.tif]

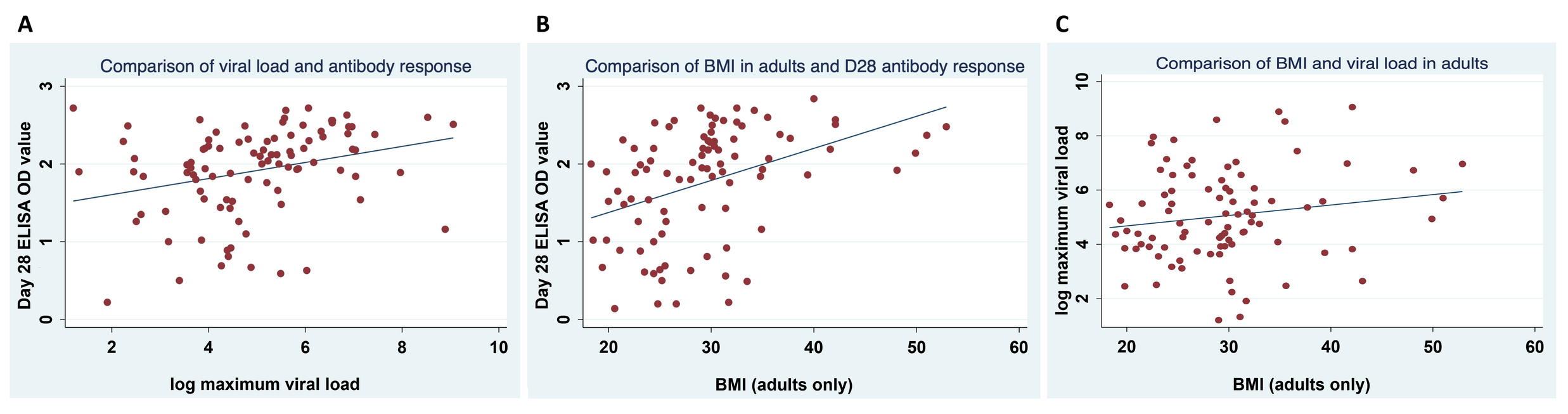

Supplement: S2 Fig — Comparison of ELISA OD value on study day 28 and maximum viral load measured by PCR testing (A) and BMI of adults (B) as well as comparison of maximum viral load and BMI of adults (C). (TIF) [file pone.0313467.s002.tif]
